# Plant hormone transporters: what we know and what we would like to know

**DOI:** 10.1186/s12915-017-0443-x

**Published:** 2017-10-25

**Authors:** Jiyoung Park, Youngsook Lee, Enrico Martinoia, Markus Geisler

**Affiliations:** 1 Division of Biological Sciences, University of California, San Diego, 9500 Gilman Drive, La Jolla, CA 92093-0116 USA; 2 Division of Integrative Bioscience and Biotechnology, POSTECH, Pohang, 37673 South Korea; 3 Institute for Plant Biology, University of Zurich, Zollikerstrasse 107, 8008 Zurich, Switzerland; 4 Department of Biology, University of Fribourg, 1700 Fribourg, Switzerland

## Abstract

Hormone transporters are crucial for plant hormone action, which is underlined by severe developmental and physiological impacts caused by their loss-of-function mutations. Here, we summarize recent knowledge on the individual roles of plant hormone transporters in local and long-distance transport. Our inventory reveals that many hormones are transported by members of distinct transporter classes, with an apparent dominance of the ATP-binding cassette (ABC) family and of the Nitrate transport1/Peptide transporter family (NPF). The current need to explore further hormone transporter regulation, their functional interaction, transport directionalities, and substrate specificities is briefly reviewed.

## Plants need many hormone transporters

The hormone concept was coined in the beginning of the 20th century, although it had been already postulated by the Darwins based on their observation that phototropic bending of coleoptiles employs a spatial separation between the place of sensing in the shoot tip and the place of bending in the shoot below [1]. Today we know that what Darwin called ‘certain influence’ [1] is the phytohormone auxin, which is synthesized in the coleoptile tip, where light is sensed, and transported down to the appropriate site of action in the shoot [2].

Since then, many plant hormones have been identified and most have been found to be synthesized at different sites from their actions. Thus, it has become evident that hormones are transported, and consequently, hormone transporters are essential for precise regulation of plant growth and development by plant hormones. Now we understand that the proper integration of environmental inputs with plant endogenous signaling requires the action of hormone transporting proteins. Evidence for this includes severe developmental and physiological impact that is caused by hormone transporter loss-of-function mutations: without transport, no correct hormone action.

Long distance transport has been demonstrated for many plant hormones, including auxins, abscisic acid (ABA), cytokinins, gibberellins (GAs), strigolactones, and salicylic acid. Hormones transported with the transpiration stream have to be loaded into the xylem and unloaded at the target cells. Similarly, hormones transported through the phloem may also require loading and unloading steps. Transporters involved in long distance transfer of auxin, ABA, cytokinins, and strigolactones have been reported, and further investigation would allow us to understand fully how individual transporters cooperate to achieve a systemic level of transport including via xylem or phloem. In cases where long-distance transport is achieved by cell-to-cell transport, such as for auxins, the highly coordinated action of import and export transporters at the contact surfaces of neighboring cells is needed. For local action of hormones (paracrine-like in animals) in some tissues such as seeds, short-distance transport between cells is sufficient, which could be carried out by exporter and importer proteins in adjacent cells (for example, see “Abscisic acid” section). Furthermore, in most cases plant hormones do not have only one target cell; therefore, several pairs of importers and exporters are required for the correct allocation and in order to guarantee the function of a complex hormonal network.

Only in a few cases has it been demonstrated that a plant hormone is synthesized in the same cells where its function is required, and these may not require intercellular transport mechanisms. A well-documented example regards ABA. Guard cells are able to synthesize ABA autonomously upon drought, thus rapidly closing stomata to prevent water loss [3]. Another case is ethylene, which is highly volatile and diffuses freely through lipid membranes. Therefore, ethylene synthesized at a specific location could be perceived by other cells far away from its site of synthesis as well as in the same cells without the need for a transporter [4]. However, it should be mentioned that the ethylene precursor ACC is mobile, and hence in this case it is not the hormone itself that is transported, but its precursor [5, 6].

Several hormones such as auxin and ABA are weak acids and hence partially present in their protonated forms in physiological pH conditions, which can diffuse quite easily through membranes [7, 8]. Therefore, originally it has been postulated that transport of auxin and ABA from the apoplast into cells would occur by diffusion through the plasma membrane. Nowadays we know that diffusion of these hormones over the plasma membrane plays only a minor role in vivo and that transporters are required for proper delivery of auxin and ABA (see “Auxin” and “Abscisic acid” sections for details).

In this review, identification of plant transporters implicated in hormone transport and their individual and overlapping roles will be discussed. The transporters range from primary active pumps belonging to the ATP binding cassette (ABC) transporter family (which couple hormone translocation to direct ATP hydrolysis); antiporters and symporters from the NITRATE TRANSPORTER (NRT) and Multidrug and toxic compound extrusion (MATE) transporter families (which use the proton motive force to create hormone concentration gradients); and facilitators of the SWEET family. This review will highlight how hormone transporters have been initially identified and—if so—how their individual activities and their substrate specificities have been verified. This would be crucial because deducing transport activities from protein sequence alignments or mutant phenotypes can have many pitfalls. For example, large substrate ranges of ABC, NRT, and MATE transporter families make it difficult to predict a specific function of a transporter in hormone transport without adequate biochemical analyses. In cases where a protein of interest functions as a modulator of other transporters, it is necessary to carefully interpret transport activities/mutant phenotypes to elucidate functions of the modulator itself. Furthermore, phenotypes of transporter mutant plants need to be carefully examined since they may reflect secondary effects, such as changes in carbohydrate or nitrogen nutrition. It should also be mentioned that, although most studies have used plants carrying stable mutations in transporter genes to investigate functions of the transporters, it cannot be excluded that the observed phenotypes may be due to an adaptation of the plant to a specific mutation. In conclusion, it is always necessary to integrate multiple sources of evidence to demonstrate hormone transport by a transporter.

Finally, we will summarize what we would like to know about hormone transporters in the near future with respect to their discrete regulation, functional interaction, transport directionality, and substrate specificity.

## What we know about plant hormone transporters

### Auxins

Natural auxins represent a surprisingly heterogeneous group of small aromatic carboxylic acids that act as a main coordinative signal for virtually all plant growth and development processes [9]. The most abundant auxin, IAA (indole-3-acetic acid), controls transcriptional regulation of multiple developmental processes via activation of a nuclear co-receptor complex (for a review, see [9]) but seems to regulate plant performance also on the post-transcriptional level [10].

**Fig. 1.**
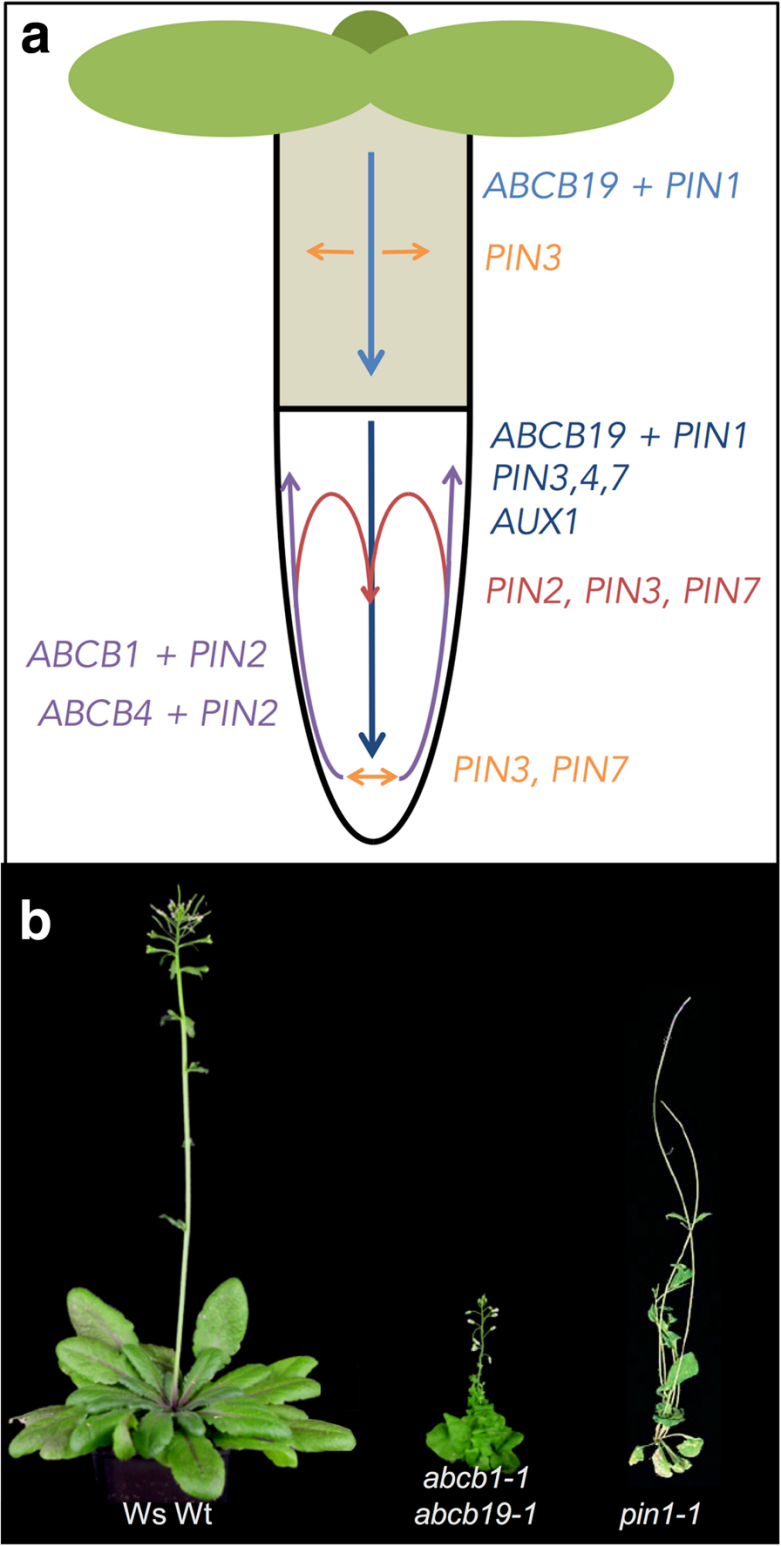
Main auxin transport routes and transporters in *Arabidopsis* seedlings and growth phenotypes of adult, mutant plants lacking auxin exporters. **a** Contribution of ABCB-, AUX1/LAX-, and PIN-type transporters in main auxin (IAA) transport routes (*arrows*) of seedling roots and hypocotyls, where auxin research has been focused. Concerted actions indicating a shared, overlapping function of two (or more) PIN- and ABCB-type auxin exporters (for details, see [12]) are designated by *color-coded arrows*. Figure modified from [12]. **b** Growth phenotypes of soil-grown plants defective in auxin export. From *left* to *right*: wild type (ecotype Wassilewskija, Ws), *abcb1-1/abcb19-1* (taken from [36], and reprinted by permission of the publisher, Taylor & Francis Ltd, http://www.tandfonline.com) and *pin1-1* (taken from [154] with permission). Note that, in contrast to the *abcb1 abcb19* double mutant, *abcb1* and *abcb19* single mutants reveal only very subtle growth phenotypes, suggesting—despite opposite main transport routes—*complementary* action by ABCB19 and ABCB1 isoforms, respectively. Also note that other single *PIN* and higher order *PIN* mutants (without *PIN1*) [28] as well as single *AUX1/LAX* mutants and the quadruple *aux1/lax* mutant [23] reveal only very subtle growth phenotypes

Auxin responses depend on local auxin concentrations that are created by combinations of auxin biosynthesis, metabolism, and transport [7, 9]. In higher plants, IAA is translocated by two pathways: first, a fast, non-directional passive transport through the phloem vasculature from biosynthetically active source tissues (such as young shoots) towards sink tissues (such as the root) [7]. Second, a slow, directional cell-to-cell transport of IAA, usually referred to as polar auxin transport (PAT), that is provided by active movement of IAA over the plasma membrane by auxin transporter proteins [9]. The directional, cell-to-cell transport is also possible for other plant hormones (such as jasmonic acids or strigolactones [11]), but has not yet been clearly demonstrated [12]. During PAT, local auxin minima and maxima, which are thought to be responsible for many developmental cues, are established, maintained, and fine-tuned by independent and combined action of members of at least three major families of auxin transporters [13] (Fig. 1): AUXIN1-RESISTANT1 (AUX1)/LIKE AUX1 (hereafter AUX1/LAX), PIN-FORMED proteins (hereafter PINs), and members of the B subfamily of ATP binding cassette (ABC) transporters (hereafter ABCBs) [14, 15]. While the *aux1* and *pin1* mutants were first identified in genetic screens for auxin (2,4-D) resistance [16] and developmental (flower) phenotypes [17], respectively, *ABCB19/MDR1/PGP19* was originally isolated in a screen for genes differentially expressed in response to the anion channel blocker NPPB (5-nitro-2-(3-phenylpropylamino)-benzoic acid) [18]. Meanwhile, different *abcb* mutants were also identified in classic genetic screens [19, 20]. For the key members of all three families, solid experimental data have been provided that characterize them as bona fide auxin transporters and their substrate specificities have been examined (reviewed in [13]).

Because IAA is a weak acid (pKa = 4.75), a portion of it is lipophilic in its protonated form at apoplastic pH (≈5.5); nevertheless, cellular uptake across the plasma membrane was shown to be dependent on AUX1/LAX proteins functioning as high-affinity auxin-proton symporters [21, 22]. AUX1 has roles in both the acropetal auxin stream in the vascular cylinder as well as in auxin exclusion from the lateral root cap in the basipetal auxin stream (Fig. 1). In protophloem root cells, AUX1 reveals an asymmetric plasma membrane polarity at the shoot-ward facing polar domain. Members of this family form a small gene family of four isoforms in *Arabidopsis* and regulate different developmental processes; however, the quadruple *aux1/lax* mutant reveals only very subtle growth phenotypes, including phyllotactic patterning defects [23].

An additional auxin uptake mechanism has been described with NITRATE TRANSPORTER 1.1 (NRT1.1), which is suggested to act as a dual function auxin transporter/nitrate transceptor under nitrogen starvation conditions [24, 25].

According to the chemiosmotic hypothesis [26, 27], auxin efflux may be a rate-limiting step in PAT [13] and efflux transporters of the PIN and the ABCB families are current candidate transporters for providing this activity. PINs, named after the pin-formed phenotype of the *pin1* loss-of-function mutant (Fig. 1), are permease-like auxin transporters. PIN transporters can be classified into “long PINs” and “short PINs” based on the presence of an extensive hydrophilic loop separating two transmembrane domains. In *Arabidopsis*, long PINs (PIN1, 2, 3, 4, and 7) function as plasma membrane exporters, while short PINs (PIN5, 6, and 8) localize to endomembrane structures [13]. Most PINs, especially PIN1 and PIN2, exhibit a remarkable polar localization aligning well with their proposed roles in PAT, which has resulted in the concept that long PINs provide the vectorial component during PAT. As such they are thought to shape the so-called “reflux loop” in the root tip [28], controlling various developmental processes.

Unlike PINs and AUX1/LAX proteins, a subgroup of ABCBs act as primary active auxin pumps that are able to transport against steep auxin gradients [9, 14, 29]. Currently, ABCB isoforms, ABCB1, 4, 14, 15, 19, and 21, have been associated with PAT; however, only for ABCB1, 4, 19, and 21 have transport activities been tightly confirmed [29–34]. Interestingly, whereas ABCB1 and ABCB19 were shown to function as specific auxin exporters, *Arabidopsis* ABCB4 and 21 [29, 30, 33, 34] and *Oryza sativa* ABCB14 [35] were suggested to function as facultative IAA importers/exporters [30].

In correlation with their identity as auxin pumps, ABCBs are mainly expressed in meristematic tissues (both root and shoot), which contain high auxin concentrations [29, 36]. These roles are on one hand in agreement with *Arabidopsis* mutant phenotypes for *abcb19* and *abcb1 abcb19*, showing epinastic leaves and dwarfism as expected for an excess of apical auxin [18, 37]. On the other hand, phenotypes are also in line with gradual reductions in auxin transport; the *abcb1 abcb19* mutant showed a 70% reduction in polar auxin transport [38]. In summary, ABCBs contribute much to polar transport of auxin (Fig. 1) despite their non-polar expression pattern [29].

Short PINs (PIN5, 6, 8) and members of a recently discovered family of PIN-LIKE (PILS) transporters were shown to function as auxin transporters on endomembrane (mainly endoplasmic reticulum (ER)) structures [39–41]. Their involvement in PAT is unclear; instead they were suggested to play roles in cellular auxin homeostasis by transporting auxin away from the nuclear auxin receptor. In another endomembrane, a tonoplast importer, WALLS ARE THIN 1 (WAT1), was shown to retrieve auxin from vacuoles, suggesting vacuoles function as an additional auxin compartment that might contribute to auxin homeostasis [42].

Several studies reported that another native auxin, indole-3-butyric acid (IBA), is also transported in a polar fashion by employing IAA-independent transport systems [43–46]. IBA hypersensitivity of mutants defective in *ABCG36/PDR8/PEN3* [46, 47] and *ABCG37*/*PDR9*/*PIS1* [45, 46, 48] suggests that IBA is a common substrate exported by both ABCG36 and ABCG37, but IBA transport activity has only been examined for ABCG37 so far [45]. The roles of the two transporters in the putative apical or basal polar IBA transport are as yet unclear since they exhibit an outward-facing lateral polar localization, instead of an apical or basal polar localization expected for contributors to polar transport, in the root epidermis.

The D subfamily member of ABC transporter, PEROXISOMAL ABC TRANSPORTER1 (PXA1/ABCD1/PED3/COMATOSE (CTS)), seems to be essential for full IBA responsiveness [49–51]. However, in contrast to fatty acyl-CoAs, IBA transport activity of ABCD1 has not yet been tested and *abcd1* mutant plants exhibit pleiotropic phenotypes, which may indicate wider substrate specificity for ABCD1 (see “Jasmonic acid” section).

In summary, in light of a pivotal role of auxin for plant performance, it does not come as a surprise that transport of auxin is distributed amongst several transporter families. While for key members of each transporter class auxin transport has been demonstrated biochemically, the contributions of individual family members in a complex auxin transport system are still not well understood.

### Abscisic acid

Abscisic acid (ABA) is important for plant responses to environmental stresses such as drought and to pathogen infection, and for the regulation of developmental processes, including germination [8, 52, 53]. When plants are exposed to water deficiency, synthesis of ABA increases greatly. Perception of ABA by receptor proteins PYRABACTIN RESISTANCE (PYR)/PYR-LIKE (PYL)/REGULATORY COMPONENTS OF ABA RECEPTOR (RCAR) in the nucleus initiates a series of signal transduction steps involving the early signaling components PROTEIN PHOSPHATASEs 2C (PP2Cs) and SUCROSE NON-FERMENTING-1-RELATED PROTEIN KINASEs 2 (SnRK2s), which enable plants to cope with drought stress [54]. The most extensively studied ABA-induced response to drought is stomatal closing [55, 56]. For a long time, roots were suspected to be the place of ABA synthesis upon drought [8, 57] in order to close stomata since, in nature, roots are the first site where the drought signal is perceived. Then ABA or its inactive form was postulated to move to the shoot via the vasculature. However, more recent reports suggest that drought-induced ABA synthesis occurs in shoot vasculature [58, 59]. Consistent with this observation, the rate-limiting enzyme for ABA synthesis, 9-*cis*-epoxycarotenoid dioxygenase 3 (NCED3), was induced in cells in vasculature in response to drought [60]. Recently, guard cells were also shown to produce ABA autonomously under low humidity [3]. A proportion of the weak acid ABA (pKa = 4.7) could diffuse into the plasma membrane from apoplastic spaces under normal conditions. However, as apoplastic pH increases under drought stress [61], the amount of the freely permeable form of ABA decreases. Therefore, ABA transporters are necessary to transfer ABA across the plasma membrane from the sites of synthesis (vascular tissues) to the sites of action (the guard cells), especially under drought conditions when ABA biosynthesis is induced.

**Fig. 2.**
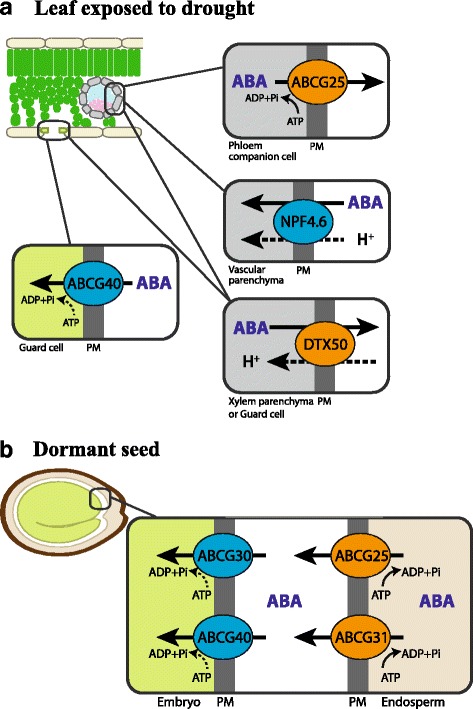
ABA transporters in the leaf exposed to drought and in the dormant seed. Transporters that mediate efflux of ABA are marked in *orange*, and transporters that mediate influx of ABA are marked in *blue*. **a** When exposed to drought, ABA synthesized in vascular parenchymal cells is exported out of the cells via ABCG25 and DTX50. NPF4.6/AIT1 is reported to regulate the level of ABA in the vascular parenchymal cells. In the guard cell, ABCG40 takes up ABA, which induces stomatal closing. **b** Concerted action of ABC transporters mediates ABA transfer from endosperm to embryo, thus maintaining seed dormancy. At the endosperm cells, ABCG25 and ABCG31 export ABA to the extracellular space. ABCG30 and ABCG40 take up ABA into embryo cells. Under normal conditions, most ABA inside the plant cell (pH around 7.5) is in anionic forms, while in the apoplast at a pH between 5 and 6 a larger part of ABA is present in uncharged forms. Under drought conditions when the pH of the xylem sap increases, more ABA in the apoplast becomes charged. Whether ABC transporters that mediate ABA import use ATP hydrolysis as an energy source is not clear (*dashed arrows*). A driving force for DTX50 and NPF4.6 was not examined, but it is most likely dependent on proton motive force, similarly to many MATE transporters and NPF family transporters (*dashed arrows*). *PM* plasma membrane

G-subfamily ATP-binding cassette (ABC) transporters ABCG25/WBC25 and ABCG40/PDR12 were reported to transport ABA over the plasma membrane out of vascular cells to the xylem, and into guard cells, respectively [62–64] (Fig. 2). ABCG25 is expressed mainly in phloem companion cells [64]. Its identity as an ABA exporter was demonstrated by transport activity assays using radiolabeled ABA and membrane vesicles isolated from insect cells expressing ABCG25 [63]. Also, ABCG25-expressing *Xenopus* oocytes accumulated lower ABA than the control oocytes over time, when incubated in an ABA-containing bath solution [65]. ABA uptake activity of ABCG40 was demonstrated in yeast cells, a tobacco Bright Yellow 2 (BY2) cell line expressing ABCG40, and mesophyll protoplast cells of loss-of-function *abcg40* mutant plants using radiolabeled ABA [62]. *abcg40* mutant plants exhibited impaired stomatal closing movement in response to ABA, which supported its role in ABA uptake into guard cells [62]. Together, these reports supported roles of the two ABCG transporters, ABCG25 and ABCG40, in supplying ABA from vascular cells to guard cells, thereby inducing stomatal closure under drought (Fig. 2).

In addition, ABCG25 and ABCG40, as well as two other *Arabidopsis* ABCG-type transporters, ABCG30 and ABCG31, were found to transport ABA from the endosperm of seed coats to the embryo, thus maintaining seed dormancy [66] (Fig. 2). Using germination assays with seed coats and embryos from different genotypes, involvement of the ABCG transporters in seed dormancy was examined [66]. Expression profiles and transport activity assays support that ABCG25 and ABCG31 catalyze the export of ABA from the endosperm, while ABCG40 and ABCG30 are responsible for the uptake of ABA into the embryo. However, transport assays using radiolabeled ABA revealed that the endosperm of the *abcg25 abcg31* double loss-of-function plants still maintained at least 60% of the transport activity [66]. The half-size ABC transporter ABCG22 is implicated in drought stress tolerance; however, the ABA transport activity of ABCG22 was not detected in independent systems, which leaves the identity of AtABCG22 as an additional ABA transporter open [67].

DTX50, a transporter of the Multidrug and toxic compound extrusion (MATE) type transporter family [68], and NITRATE TRANSPORT1/PEPTIDE TRANSPORTER FAMILY (NPF) 4.6/NRT1.2/ABA-IMPORTING TRANSPORTER (AIT) 1, a member of the NPF transporters, have been shown to transport ABA [69] (Fig. 2). DTX50 is expressed in vascular tissues and localized at the plasma membrane. Its ABA efflux activity was examined in DTX50-expressing *Escherichia coli* cells and *Xenopus* oocytes, and mesophyll protoplasts of the *dtx50* loss-of-function mutants using radiolabeled ABA. *dtx50* mutant plants were hypersensitive to ABA in germination, root growth, and stomatal closing [68], altogether suggesting the involvement of *DTX50* in ABA-related developmental processes. NPF4.6/AIT1 was discovered in a screen for proteins that induce ABA-dependent interaction between the ABA receptor PYR1 and PP2C ABI1 in a yeast two-hybrid (Y2H) assay [69]. Transport assays and subsequent LC-MS/MS analysis supported the ABA uptake activity of NPF4.6/AIT1 in yeast. Loss-of-function mutants of *NPF4.6/AIT1* were less sensitive to exogenously applied ABA than the wild type, exhibiting a decreased inhibition of seed germination [69]. Using the same Y2H assay, other NPF members were also isolated as having potential ABA transport activity [69, 70], although their *in planta* functions remain to be further investigated.

ABA is stored in its inactive storage form, ABA-glucose ester (ABA-GE), in the vacuole and ER, and under stress conditions ABA-GE is cleaved into ABA and ABA is released into the cytosol [8, 71–73]. Uptake of ABA-GE into vacuoles has been shown to be catalyzed by AtABCC2 and a so-far-unknown proton antiporter [74]. However, transporters that sequester ABA-GE into the ER and transporters that export ABA released by glucosidases from the ER and vacuole are as yet unknown.

In short, members of many different transporter families have been found to participate in transport of ABA to cells near and far from the cells, which synthesize the hormone. Still, we expect additional ABA transporters will be identified to play critical physiological functions.

### Cytokinin

Cytokinins represent a heterogenous group of N^6^-substituted adenine derivative hormones that regulate plant growth and development, including cambial meristem activities [75]. Free-base cytokinins such as *trans*-zeatin (*t*Z) and N^6^-(Δ^2^-isopentenyl) adenine (iP), are considered active, while their nucleoside conjugates are not. Sugar conjugates including cytokinin O-glucoside are thought to be inactive storage forms [75]. Once cytokinin is perceived by receptor proteins at the plasma membrane, a phospho-relay is initiated from *Arabidopsis* His kinases (AHKs), which transfer phosphoryl groups to *Arabidopsis* His Phosphotransfer proteins (AHPs), and then to *Arabidopsis* response regulators (ARRs), thus resulting in transcriptional and posttranscriptional regulation [76].

**Fig. 3.**
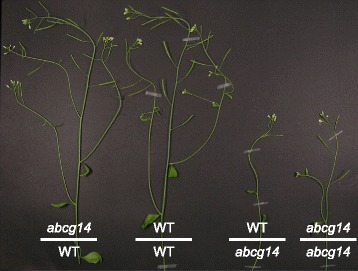
Retarded growth phenotype of cytokinin transporter mutant *abcg14*. Reciprocal grafting experiments support a role of ABCG14 in root-to-shoot transport of cytokinin, which is crucial for normal growth and development of the shoot. The graft between an *abcg14* mutant scion and wild-type rootstock (*abcg14*/WT) complemented the stunted growth of *abcg14* loss-of-function mutant plants (as shown by the graft between *abcg14* scion and rootstock (*abcg14*/*abcg14*)), while the graft between a wild-type scion and *abcg14* rootstock (WT/*abcg14*) maintained impaired growth. Image taken from [80]

Different types of cytokinins are synthesized in roots and shoots, respectively; root-derived *t*Z-type cytokinins and shoot-derived iP-type cytokinins move acropetally and basipetally, respectively, through the xylem and phloem [77, 78]. However, the molecular identity of the factors involved in long-distance transport of cytokinins remained obscure for a long time. Recently, two independent laboratories identified a G-type ABC transporter, ABCG14, as critical for acropetal transport activity of *t*Z-type cytokinins [79, 80]. *ABCG14* loss-of-function mutant plants exhibited growth retardation in shoots including small leaves and short inflorescence stems, and a reduced number of vascular bundles, rescued by exogenous supplementation of *t*Z. Reciprocal grafting experiments between *abcg14* mutant plants and isogenic wild-type plants revealed that the roots of *abcg14* cause growth retardation [79, 80] (Fig. 3). ABCG14 is expressed in cells of the root vasculature, including pericycle and procambial cells. Detailed cytokinin profiling in shoots and roots revealed that *t*Z-type cytokinin concentrations were reduced in the shoot of *abcg14* mutant plants [79, 80]. Finally, the role of ABCG14 in root-to-shoot transport of cytokinins was proven by translocation experiments using isotope-labeled *t*Z; *abcg14* plants were found to accumulate less isotope-labeled *t*Z in their shoots when fed to the roots [79, 80]. However, since ABCG14 has not been shown to transport cytokinin in any heterologous system, it is not known yet whether ABCG14 alone is sufficient for cytokinin transport or a partner protein is necessary for the function, and which kind of the many cytokinins is transported by ABCG14.

The equilibrative nucleoside transporter (ENT) family [77, 81, 82] and the purine permease (PUP) family [83, 84] were also suggested to function as cytokinin transporters. The uptake transport activity of several ENT family members, including rice ENT2 and *Arabidopsis* ENT3, ENT6, ENT7, and ENT8, was shown upon expression in yeast using radio-labeled iP-riboside (iPR) and/or *t*Z-riboside (*t*ZR) [77, 81, 82]. However, *ent3* and *ent8* mutant plants exhibited only a marginal reduction in sensitivity to both iPR and *t*ZR [82]; thus, their roles in cytokinin transport *in planta* remain obscure.

Recently, using a synthetic fluorescent reporter for cytokinin, PUP14 was isolated as a cytokinin uptake transporter at the plasma membrane and found to be important for normal development of the embryo in *Arabidopsis* [84]. Reducing *PUP14* expression activated cytokinin signal transduction in the embryo and shoot apical meristem, while *PUP14* over-expression led to inactivation of the pathway. The uptake activity of PUP14 was supported by radiolabeled *t*Z transport assays using mesophyll protoplasts expressing PUP14 and membrane vesicles isolated from PUP14-expressing tobacco plants. It was thus proposed that PUP14 takes up bioactive cytokinins from the apoplast and inhibits binding of cytokinins to plasma membrane-localized cytokinin receptors, suggesting PUP14 as a terminator of a cytokinin signal transduction [84]. This new concept for a transporter functioning in hormone depletion is, at first view, surprising since cytokinin receptors were recently shown to be enriched at the ER membrane [85]. Thus, deeper investigations would be required to establish further the proposed transporter–receptor interplay. In the case of other PUP members, such as PUP1 and PUP2, the uptake transport activity for *t*Z and iP was examined in yeast [83]; however, no genetic or biochemical evidence supporting their roles *in planta* has yet been provided. In short, an ABC transporter, ABCG14, and several ENT members have been shown to participate in cytokinin transport. Further biochemical analyses of ABCG14 and further analyses of in vivo functions of ENT members are necessary to establish a complete picture of cytokinin transport in plants.

### Gibberellins

Gibberellins (GAs) regulate germination, growth—including shoot and root elongation—and reproductive development [86, 87]. They are a hormone group of tetracyclic diterpenoid acids labeled with “A numbers” in the order they were identified. Among more than 100 GAs, only a handful are known to be bioactive and recognized by the soluble receptor protein GIBBERELLIN INSENSITIVE DWARF 1 (GID1) and negative regulator DELLA proteins [86, 87]. Reports using labeled GA provided physiological evidence that GA is translocated between different tissues ([88] and references therein). Recently, various strategies have allowed the identification of GA transporters.

Multiple members of the NPF family exhibit GA transport activities. A screen for mutant plants defective in root accumulation of fluorescein-tagged GA (GA-Fls) identified NPF3.1 as a putative GA transporter [89]. GA transport activity of NPF3.1 was examined in *Xenopus* oocytes using GA-Fls and GAs with subsequent LC-MS analyses. NPF3.1 was shown to import GA_4_ and, to a lesser extent, also GA_1_ and GA_3_ in a pH-dependent manner, consistent with the proton motive force-dependence of the NPF transporter family. *NPF3.1* loss-of-function plants did not exhibit GA-related phenotypes [89], perhaps due to functional redundancy.

Based on a Y2H screen using the GA receptor GID1a and DELLA protein GA INSENSITIVE (GAI) as a read-out, NPF4.1/AIT3 was shown to exhibit transport activities for GA_1_, GA_3_, and GA_4_ [70]. Also, using the same screening system, members of the sugar transporter SWEET family, AtSWEET13 and AtSWEET14, were identified to be involved in GA uptake [90]. Transport activities of SWEET13 and SWEET14 to GA_3_ and other GAs were further examined in yeast and *Xenopus* oocytes. SWEET-expressing yeast cells and *Xenopus* oocytes accumulated more GAs when measured by mass spectrometry. The *sweet13 sweet14* double mutant, but not the respective single mutants, exhibited reduced fertility and delayed anther dehiscence, which is restored by addition of GA_3_ [90].

**Fig. 4.**
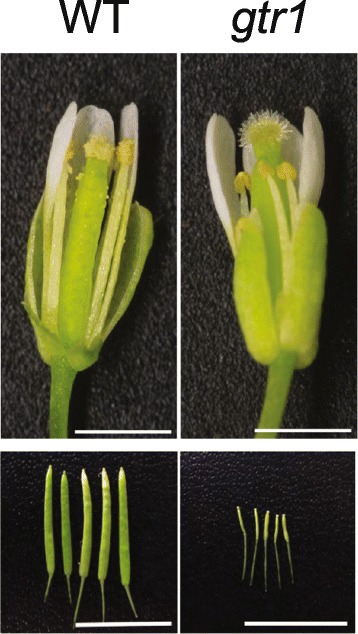
Reproductive organ phenotypes of GA/JA-Ile transporter mutant *gtr1*. Stamen growth is defective (*top panel*) and seed production is reduced (*bottom panel*) in the *gtr1* mutant. Scale bar, 1 mm (*top panel*) and 10 mm (*bottom panel*). Images taken from [92]

NPF2.10/GTR1 was reported for its role in transporting glucosinolates [91], defense compounds mainly found in the family Brassicaceae, but was also recently found to exhibit transport activities to GA and JA-Ile [92] (see also “Jasmonic acid” section). NPF2.10/GTR1 is involved in GA_3_ import, but not in the transport of the other bioactive GAs, GA_1_ and GA_4_, when examined in *Xenopus* oocytes [92]. *NPF2.10/GTR1* loss-of-function plants exhibited a defect in stamen development, and GA_3_ rescued this phenotype, suggesting that the decreased fertility of *npf2.10/gtr1* is due to defects in GA transport [92] (Fig. 4). In summary, identification of many GA transporters was possible using novel screening strategies, which overcame the limitation of the conventional strategy of observing phenotypes of single gene knockout mutants. Such new screening methods are particularly important for plant hormone transporter research since there are likely many hormone transporters with similar and overlapping functions.

### Strigolactones

Strigolactones were originally discovered as germination stimulants for parasitic weeds in the 1960s [93]. About 40 years after their discovery, scientists realized that these carotenoid-derived compounds act both as signaling compounds inducing mycorrhizal symbiosis and phytohormones exhibiting intrinsic signaling functions. Within the plant, strigolactones act as inhibitors of lateral bud outgrowth and induce lateral root initiation and procambial division, which in principle overlap with auxin actions in developmental processes above- and below-ground. A plausible explanation for the overlap was found with the discovery that auxin transport was directly controlled by strigolactones [94]. In the presence of strigolactones, an F-box protein (MAX2 in *Arabidopsis*) interacts with an α/β-hydrolase (D14 in *Arabidopsis*) and induces a downstream signaling cascade [95].

Strigolactones are produced both in roots and shoots, but grafting experiments have shown that root-derived strigolactones can rescue the shoot phenotype of a strigolactone biosynthesis mutant, indicating that strigolactones can be transported from the root to the shoot, most likely via the xylem [94]. However, whether a transporter is required for the root-to-shoot movement of strigolactones remained unknown until a recent report on the G-type ABC transporter, PDR1.

In a targeted approach, Kretzschmar *et al*. [96] identified PDR1 as a strigolactone transporter in *Petunia. Petunia axillaris PDR1* (*PaPDR1*) was localized to the plasma membrane of the root tip and hypodermal passage cells, the latter being entry points of mycorrhizal fungi. The localization of PDR1 corresponds to the observation that strigolactones are excreted from roots to induce hyphal branching of mycorrhizal fungi. *PDR1* loss-of-function plants excreted less strigolactone, were impaired in mycorrhization, and produced more branches in the aerial part of the plant. Further studies showed that the transfer of exogenously applied GR24, an artificial strigolactone, from the root to the shoot was impaired in *PDR1* mutant plants, suggesting a role of PDR1 in root to shoot transport of strigolactones [96]. The dual role of PDR1 in transferring strigolactones to the shoot and to the rhizosphere is thought to be connected with its dual polar localization: in the root tip and the cortex the transporter is apically polar localized, while in hypodermal passage cells, PDR1 is localized laterally [97]. A subsequent study in tobacco showed that *Nicotiana tabacum* PDR6 (NtPDR6), a close homologue of PaPDR1, is also localized to hypodermal passage cells and that plants expressing antisense NtPDR6 were bushier than wild type, which is in line with a reduced allocation of strigolactones to the shoot [98, 99]. In *Arabidopsis*, no PDR1 homolog or strigolactone transporter has been identified so far, despite the fact that root to shoot translocation of strigolactones has been shown in *Arabidopsis* [100]. This may be due to the fact that in *Arabidopsis* the strigolactone-like compound methyl carlactonoate performs strigolactone-attributed functions [101]. It will be interesting, therefore, to unveil the corresponding transporter for methyl carlactonoate in *Arabidopsis*, including investigation of whether this transporter shows polar localization. Strigolactone transporters are the rare examples of hormone transporters first identified from non-*Arabidopsis* plants, and they seem to be important for plant interactions with other plants and microbes in the environment. Thus, it would be interesting to study evolution of the strigolactone transporters—how they diversified during establishment of parasitism and symbiosis.

### Jasmonates

Jasmonates (JAs), the major component of the jasmine scent, are plant hormones important for reproductive development, wound responses, and plant immune signaling, especially against herbivores [102, 103]. JAs are produced via the oxidative metabolism of polysaturated fatty acids. JA biosynthesis is initiated in chloroplasts and plastids, and the subsequent steps occur in the peroxisomes. An amino acid conjugate of JAs, jasmonoyl-isoleucine (JA-Ile), is considered as the major bioactive form of JAs at the molecular level. JA-Ile is recognized by the nuclear receptor CORONATINE INSENSITIVE 1 (COI1), which then mediates proteasome-dependent degradation of the negative regulator JASMONATE-ZIM-DOMAIN (JAZ) proteins [102]. Recent reports using grafting and isotope-labeling experiments suggest that JAs or JA precursors could translocate from wounded tissues to unwounded tissues [11, 104], supporting transporter-mediated translocation of JAs (also reviewed in [105]).

Several members of NPFs were suggested to function as JA transporters. Via a Y2H assay that detects JA-dependent binding of the JA receptor COI1 and its interacting protein JAZ3, NPFs were tested for JA transport activities [70]. NPF4.1/AIT3 exhibited JA-Ile transport activity [70]; however, its *in planta* role in JA transport remains to be further elucidated due to potential redundancy with other JA transporters. Another NPF member, NPF2.10/GTR1, exhibited uptake activity for JA-Ile and JA when expressed in *Xenopus* oocytes [92]. NPF2.10/GTR1 also exhibits transport activities for other substrates including glucosinolates [91] and GA [92] (see “Gibberellin” section). *GTR1* loss-of-function mutant was impaired in transferring JA and JA-Ile from wounded leaves to undamaged ones when isotope-labeled JA and JA-Ile were fed to the wounded leaves [106].

Synthesis of the precursor form of JA, 12-oxo-phytodienoic acid (OPDA), occurs in chloroplasts and plastids, while the final step of JA synthesis occurs in the peroxisome [102]. Chemical analyses and complementation of a yeast mutant support the idea that the peroxisomal D-type ABC transporter, ABCD1/CTS/PXA1, mediates the transport of OPDA into the peroxisome and thus participates in JA biosynthesis [107–110]. When expressed in the yeast mutant lacking its homologs *PXA1* and *PXA2*, functional AtABCD1 was targeted to yeast peroxisomes and complemented yeast that is defective in β-oxidation of fatty acids [110]. Basal levels of JA and wounding-inducible JA synthesis were reduced in *abcd1* mutant plants [107, 109]. In addition, *abcd1* mutant plants exhibit pleiotropic phenotypes, including enhanced seed dormancy, defects in seedling establishment in the absence of exogenous carbon source, and resistance to indole butyric acid (IBA) and 2,4-dichlorophenoxybutyric acid (2,4-DB), which suggest diverse roles of ABCD1 in mediating transport of fatty acyl-CoAs and IBA into the peroxisome for β-oxidation [107–109, 111] (also see “Auxin” section). Two barley ABCD proteins were suggested to act as homologs of *Arabidopsis* ABCD1; HvABCD2 was shown to complement Arabidopsis *abcd1* mutant plants for OPDA sensitivity, whereas HvABCD1 complements yeast *pxa1 pxa2* for β-oxidation of fatty acids [112], suggesting they have roles in OPDA transport and JA biosynthesis [112].

JA-Ile is recognized by the nuclear COI1/JAZ co-receptor complex, which reveals the necessity for nuclear entry of JA-Ile. AtABCG16/Jasmonate Transporter 1 (JAT1) exhibits a dual localization at the nuclear envelope and plasma membrane and was shown to mediate nuclear influx of JA-Ile and cellular efflux of JA across the plasma membrane [113]. JA transport activity of ABCG16/JAT1 was verified using isolated nuclei and suspension cells from *ABCG16/JAT1* loss-of-function lines and in yeast cells pre-loaded with ^3^H-JA. Loss-of-function mutant *abcg16/jat1* exhibited compromised JA signaling [113]. Recent identification of the transporters for intracellular transfer of JA and its precursor, as well as the JA transport GTR1, confirms the importance of coordinated distribution of JA and the precursors in the biosynthesis and function of JA.

### Salicylic acid

Salicylic acid (SA) has been popular for its synthetic derivative aspirin, which is widely used for pain relief. SA is crucial for plants to invoke defense mechanisms against pathogens, especially plant innate immunity and systemic acquired resistance (SAR) [114]. Biosynthesis of SA occurs in the chloroplast, while its perception by receptor complexes is in the nucleus [115, 116]. SA is also stored in the vacuole as an inactive form, SA O-β-glucoside (SAG) [114], altogether suggesting the necessity of transporters that mediate intracellular SA transport.

*ENHANCED DISEASE SUSCEPTIBILITY5* (*EDS5*)/*SA INDUCTION-DEFICIENT1* (*sid1*) was isolated from early screenings for enhanced pathogen susceptibility and defective SA accumulation after pathogen infection [117, 118]. A recent report elucidated that *EDS5* encodes a MATE-type transporter localized at the chloroplast envelope that exports SA from the chloroplast to the cytoplasm [119]. SA transport activity of EDS5 was demonstrated in isolated chloroplasts and heterologous yeast system incubated with radio-labeled SA [119], although more rigorous biochemical analysis will be needed to confirm that the direction of SA transport by EDS5 is out of the chloroplast.

Transport of an inactive storage form of SA, SA O-β-glucoside (SAG), into the vacuole is dependent on ATP and is thus suggested to require active transport, such as an ABC transporter-type mechanism in soybean [120, 121]. Also the well-known systemic distribution of SA or related mobile signals that induce systemic acquired resistance (SAR) ([114] and references therein) is dependent on a dedicated transport system. However, in both cases the identity of the transport systems has remained elusive.

### Ethylene

Ethylene is a gaseous hormone involved in many physiological processes, including fruit ripening, senescence, abscission, and immune signaling, and also well known for crosstalk with other hormones including auxin and JA [122]. Ethylene is known to be freely diffusible through membranes and is thus able to move between cells and the intracellular space without transporter proteins [4]. However, the immediate precursor of ethylene, 1-aminocyclopropane-1-carboxylic acid (ACC), has been reported as a non-gaseous mobile signal, including long-distance transport through the xylem and the phloem ([5, 6] and references therein).

*ACC-resistant2* (*ARE2*) was isolated for insensitivity of the mutant plant to exogenously applied ACC, although its responses to ethylene were not altered [123]*. ARE2* encodes for LYSINE HISTIDINE TRANSPORTER1 (LHT1), a member of the amino acid transporter family [123]*.* Transport assays using ^14^C-ACC in protoplasts from *lht1* mutant plants supported ACC uptake activity of a plasma membrane localized protein LHT1 [123]. LHT1 would thus provide a starting point to unveil the mechanism of ACC transport and the regulation of ethylene responses.

The ACC conjugate derivative N-malonylACC (MACC) is synthesized in the cytosol and transported into the vacuole for storage in an ATP-dependent manner [124]. The molecular identity of the MACC transporters still remains to be elucidated.

### Other hormones

Brassinosteroids (BRs), with their well-known growth-promoting functions, have been thoroughly studied in terms of biosynthesis and signaling [125, 126]. Nevertheless, transport of BRs has not been unveiled much thus far. BRs are generally thought not to be transported between and within organs [127, 128]. However, they are synthesized inside the cell, and their receptors are localized at the plasma membrane, exposing their ligand-binding sites to the extracellular space [125]. Therefore, the movement of BRs from the inside to the outside of the cell is necessary to initiate BR signal transduction and might thus—despite membrane permeability of these sterol compounds—require a plasma membrane exporter. Thus, it would be worth examining whether BR transport is mediated by transporter proteins.

Florigen is a hormone-like molecule responsible for inducing flowering, and its molecular identity was long sought after [129, 130]. Florigen is translocated from its site of synthesis, the leaf, to the shoot apex [129, 130]. FLOWERING LOCUS T (FT) and FT-like proteins are suggested to fulfill the properties for florigen. They are produced in leaves and transported via the phloem to the shoot apical meristem of buds and growing tips [131]. Transport of FT into phloem companion cells was reported to be mediated by FT-INTERACTING PROTEIN1 (FTIP1) [132]. FTIP1 was shown to interact with FT in phloem companion cells and to control movement of FT from phloem companion cells to sieve elements, as supported by immunogold labeling of FT proteins *in planta* [132]. However, the transport mechanism for FTIP1 still remains elusive.

Small peptides are thought to have hormone-like activities that are involved in cell-to-cell signaling, and play crucial roles in plant growth and development, including defense mechanisms, the control of cell division and expansion, and pollen self-incompatibility [133]. Most of these small peptides are likely to be secreted to the apoplast, and thus transporters for these peptides might exist, especially when peptide hormones function in distal cells.

## What we would like to know about plant hormone transporters

Our inventory of hormone transporters reveals that many hormones are transported by members of distinct transporter classes, such as primary active ABC transporters, secondary active antiporters/symporters, and facilitators. What is remarkable is the apparent dominance of ABC transporters, mainly of the B and G subfamilies transporting auxins, ABA, cytokinins, strigolactones, and JA, and of the NPF transporting GA, ABA, and JA. However, we do not know yet why these groups of transporters are particularly more suitable for transport of plant hormones than other types. The reason may be that these transporter families accept chemically and structurally different classes of compounds and have evolved to recognize hormones. Another large transporter family transporting various substrates is the MATEs, for which transport of salicylic acid and ABA has been postulated. A large number of MATE transporters have not been functionally characterized yet, most likely due to their functional redundancy. Also, approximately 800 secondary transporters are predicted in *Arabidopsis* and rice and many of them await characterization [134].

At first glance it is surprising that one hormone can be imported or exported by several transporters, often belonging to different classes. For auxin transporters it has been suggested that their energization (primary vs. secondary active) correlates with different expression and polarity profiles and thus seems to match distinct functions in dependence with local hormone concentrations. Another aspect to be considered is that primary active transporters that are driven directly by ATP hydrolysis are likely to transport much smaller numbers of substrates per unit of time than the secondary active transporters. Thus, the primary active transporters may play a role in fine-tuning of hormone levels (as has been suggested for ABCB-type auxin transporters), while secondary active ones could function in mass transport of hormone molecules (as has been suggested for PINs), although this has not been demonstrated in detail for either auxin transport or other hormones. However, for auxin transporters, this possibility is supported by independent evolution of the different transporter families: ABCBs are evolutionarily conserved from ancient organisms, while PINs apparently appeared with the first land plants [135], arguing for involvement of PINs in the creation of more advanced plant architecture and performance. The appearance of land plants would have required the faster establishment of auxin gradients, as can only be provided by secondary active transporters (PINs) that usually show a higher turnover number. Interestingly, one study reported cooperative action of ABCB-PIN pairs [12] resulting in synergistic (activation) or antagonistic (inhibition) interactions between the two types of auxin transporters [38]; it will be interesting to investigate if similar mechanisms are also found for other hormone transporters. Altogether, it appears that, in plants, concerted actions of distinct classes of hormone transporters—which function as fine-tuning transporters, mass transporters, and/or backup transporters—are a prerequisite for their appropriate function in hormone transport.

Interestingly, some members of B and G subfamilies of ABC transporters function as hormone importers. *Arabidopsis* ABCG30 and ABCG40 import ABA [62, 66], whereas ABCB4 and ABCB21 [30, 33, 34] and rice ABCB14 [35] were suggested to function as auxin importers. This is surprising since most of the eukaryotic ABC proteins were found to be exporters [12]. Recently, evidence was provided that ABCB4 and ABCB21 are facultative IAA importers/exporters whose transport directionality is determined by intracellular auxin concentrations [30]. Obviously, any enzyme can function both ways; however, at the moment it is unclear if the hormone importers function as strict ATPases or as ATP-regulated channels. Mammalian ABCC7/CFTR (Cystic fibrosis transmembrane conductance regulator) was shown to require ATP for channel opening during chloride export [136].

With a few prominent exceptions, surprisingly little is known about the posttranscriptional regulation of hormone transporters in general. Establishment of PIN polarity has been studied extensively and is thought to be provided by dynamic, clathrin-mediated internalization [137]. PIN polarity is regulated by the combined action of the serine/threonine protein kinase of the AGC family, PINOID, and the protein phosphatase 2A, PP2A, which modulates the phosphorylation status of the hydrophilic PIN loop [138]. The plasma membrane presence of ABCB1, 4, and 19 is dependent on the action of the FKBP42 protein, TWISTED DWARF1 (TWD1)/ULTRACURVATA2 (UCU2), functioning as a chaperone during ER to plasma membrane delivery [139, 140]. AUX1/LAX proteins appear to employ a distinct subcellular targeting mechanism from PIN and ABCB type auxin exporters [137, 141]. Recently, it was shown that lateral polarity of ABCG36 is dependent on ACTIN7 and the exocyst tethering complex [142]. Petunia PDR1 was reported to have dual polar localization: apically polar localized in the root tip and the cortex, and laterally localized in hypodermal passage cells [97]. In this respect, it is worth mentioning that despite directional delivery of many plant hormones, until now, polarized localization of hormone transporters has been demonstrated only for auxin and strigolactones. More detailed analyses might provide similar evidence for other hormone transporters which are involved in directional hormone transport.

Excitingly, some hormones seem to promote their own signal by stabilizing the plasma membrane presence of their transporters: in the case of ABCG25 [143] and PIN1 [144], localization of the proteins at the plasma membrane was found to be induced upon application of exogenous ABA and auxin, respectively. Despite the fact that underlying mechanisms of hormone-induced plasma membrane localization are different for ABCG25 (activation of recycling from early endosomes) and PIN1 (inhibition of endocytosis), it might be worth investigating whether plasma membrane stabilization of the transporters by the hormone substrates is a conserved phenomenon.

Protein phosphorylation regulates not only protein targeting of hormone transporters, but also their transport activity. Phosphorylation of cytoplasmic PIN loops by PINOID and related D6 protein kinases was recently shown to be essential for PIN transport activity [145]. Transport activity of ABCB1 and ABCB19 was also regulated by protein phosphorylation by PINOID, and the blue light receptor, PHOTOTROPIN1, respectively [146, 147]. Transport activity of GTR1 in *Xenopus* oocytes was abolished when a threonine residue between transmembrane 3 and 4 was substituted to the phospho-mimicking aspartic acid, and it was suggested that de-phosphorylation of the threonine is important for dimerization of GTR1 and plasma membrane localization [148]. Whether protein phosphorylation involves regulation of other hormone transporters would be interesting to investigate in the future.

Another exciting finding is that plant hormone transporters, at least a subset of ABC-type hormone transporters, have a very high degree of substrate specificity in contrast to their mammalian orthologues: ABCG25 and ABCG40 seem to transport only the physiologically active ABA enantiomer, (S)-ABA, but not (R)-ABA [62, 63], while ABCB1 is specific for a subset of auxins, including the synthetic, active auxin 1-NAA, but does not transport 2-NAA [149]. The molecular basis for this high specificity is unknown. However, kingdom-specific, putative auxin-binding motif clusters were recently found in the transmembrane domains of ABCB1 [150], which await biochemical characterization to confirm predicted function.

In contrast, some members of ABC transporters seem to have multi-substrate specificity: *abcd1/cts* mutant plants exhibit pleiotropic phenotypes, including increased seed dormancy, defects in seedling establishment without an additional carbon source, and resistance to IBA, 2,4-DB, and OPDA, which suggests diverse roles of ABCD1 in mediating transport of fatty acyl-CoAs, IBA, and OPDA into the peroxisome [49, 51, 112, 151]. Further, ABCG36 and ABCG37 transport structurally unrelated auxinic compounds like IBA, 2.4-D, and NPA [45, 152], but not IAA [45], while ABCG36 seems to also transport the heavy metal cadmium [153] and indole glucosinolates [47]. Finally, NPF2.10/GTR1 has transport activities for diverse substrates, including GA_3_, JAs, and glucosinolates, which correspond to pleiotropic phenotypes of the loss-of-function mutant plant [91, 92]. More rigorous biochemical verification of substrate specificities could clarify whether the proposed multi-substrate specificity can be safely attributed to individual transporters. In-depth biochemistry combined with physiological experiments would also uncover whether multi-specificity is linked to specific localization of the transporters *in planta*, or regulated by posttranslational modification and/or interacting partners.

## Conclusions

In summary, it appears that we have learned a lot about plant hormone transport over past years by identifying individual roles of transporters and their impact on hormone physiology. This review underlines the central roles of hormone transporters for hormone action and thus proper plant growth, architecture, and survival. However, many questions remain to be answered about their discrete regulation, functional interaction, transport directionality, and substrate specificity. Finally, many hormones, like IAA and ABA, are demobilized and deactivated by conjugation. However, besides the physiological function and conversion of these conjugates, their transporters are mostly unknown.
